# Supplementary figures and images for: Diabetes affects the composition of the respiratory tract microbiome and transcriptome in patients with viral pneumonia
Source: Microbiol Spectr. 2026 Apr 6;14(5):e01911-25. doi: 10.1128/spectrum.01911-25 (PMC13141871; doi:10.1128/spectrum.01911-25)

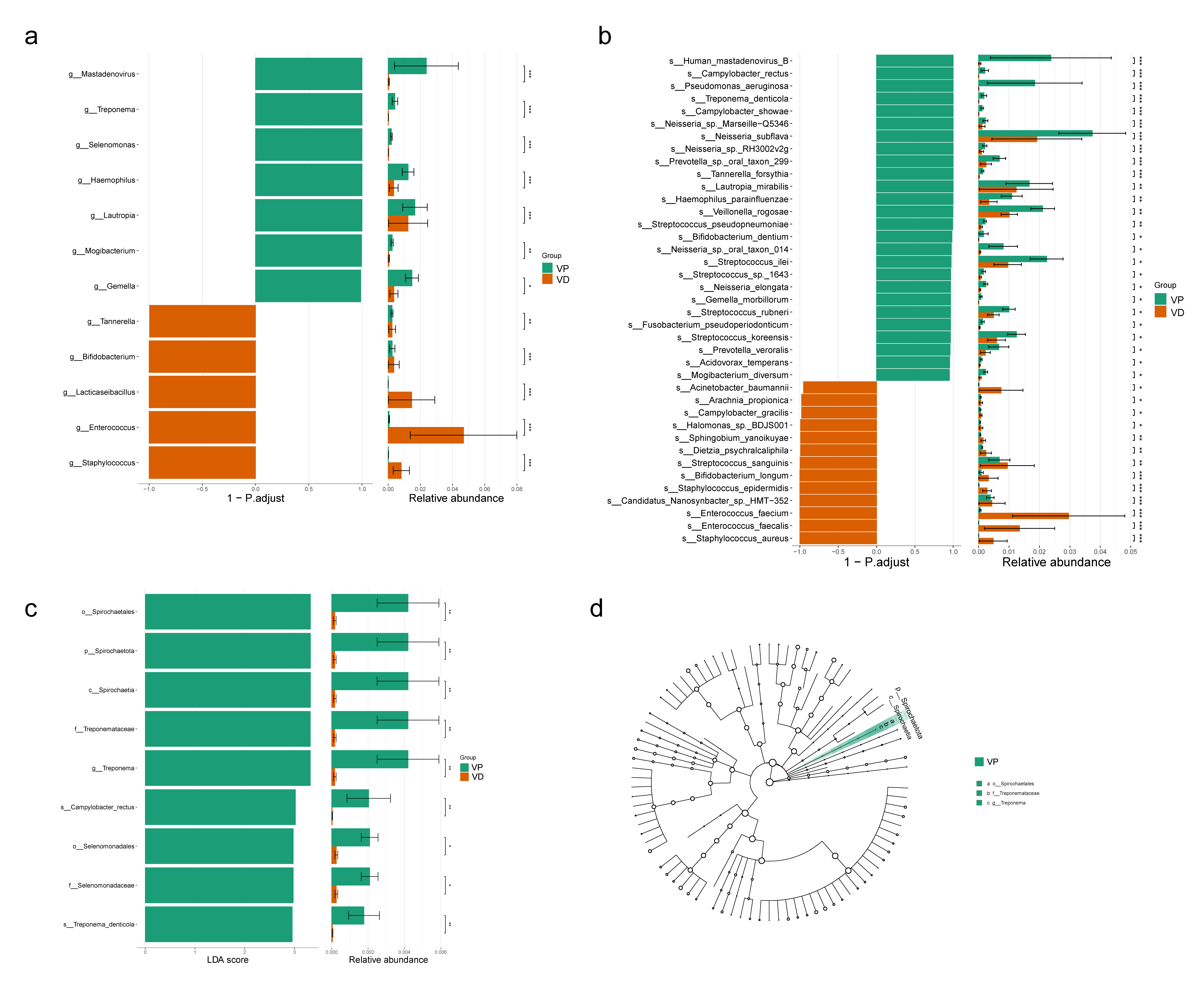

Supplement: Figure S1 — Differences in microorganisms between VD and VP Groups. [file spectrum.01911-25-s0001.tif]
