# Supplementary material for: Diabetes affects the composition of the respiratory tract microbiome and transcriptome in patients with viral pneumonia
Source: Microbiol Spectr. 2026 Apr 6;14(5):e01911-25. doi: 10.1128/spectrum.01911-25 (PMC13141871; doi:10.1128/spectrum.01911-25)
Supplement: Supplemental legends — Legends for Tables S1 to S7 and Figure S1. [file spectrum.01911-25-s0002.docx]

**Supplementary Figure and Tables Legend**

Figure S1 Differences in Microorganisms between VD and VP Groups. Differences in microbial species between VD and VP groups at the genus (a) and species (b) level analyzed by DEseq2. Bar chart (c) and evolutionary branching diagram (d) of microbial differences between VD and VP groups analyzed by LEfSe.

Table S1 Kingdom-level species abundance table.

Table S2 Phylum-level species abundance table.

Table S3 Class-level species abundance table.

Table S4 Order-level species abundance table.

Table S5 Family-level species abundance table.

Table S6 Genus-level species abundance table.

Table S7 Species-level species abundance table.
